# Developing Country-Specific Question Prompt Lists About Dementia Palliative Care for Family Caregivers

**DOI:** 10.1093/geroni/igaa057.2846

**Published:** 2020-12-16

**Authors:** Laura Bavelaar, Jenny van der Steen, Maria Nicula, Sophie Morris, Sharon Kaasalainen, Wilco Achterberg, Kevin Brazil

**Affiliations:** 1 Leiden University Medical Center, Leiden, Zuid-Holland, Netherlands; 2 McMaster University, School of Nursing, Hamilton, Ontario, Canada; 3 Queen’s University Belfast, School of Nursing and Midwifery, Belfast, Northern Ireland, United Kingdom; 4 McMaster University, Hamilton, Ontario, Canada

## Abstract

We aimed to develop question prompt lists for family caregivers of nursing home residents with advanced dementia to augment advance care planning conversations. In the context of a joint European-Canadian study, we used standardized nominal group methods to create country-specific lists of questions. (Bereaved) family caregivers of persons with dementia read an information booklet about end-of-life care for people with dementia, and generated questions to ask healthcare professionals. They also marked the most important questions from pre-selected questions from other lists. In the Netherlands, 20 participants contributed to a question prompt list of 24 questions that gravitated towards questions about terminating life and the responsibilities of physicians and family involved in decision making. In Canada, 4 participants came up with a question prompt list of 15 questions, related mostly to staff-family communication, with some the same as selected in the Netherlands. Data from the other countries will be presented too.

